# The lactic acid bacteria metabolite phenyllactic acid inhibits both radial growth and sporulation of filamentous fungi

**DOI:** 10.1186/1756-0500-6-464

**Published:** 2013-11-14

**Authors:** Åsa Svanström, Silvio Boveri, Emma Boström, Petter Melin

**Affiliations:** 1 Uppsala BioCenter, Department of Microbiology, Swedish University of Agricultural Sciences, P.O. Box 7025, Uppsala SE-750 07, Sweden; 2 Present address: Department of Agricultural and Food Sciences, University of Modena and Reggio Emilia, Via Amendola, Reggio Emilia 2-42100, Italy

**Keywords:** *Aspergillus niger*, Conidia, *Lactobacillus*, Dairy products, Sourdough, *phiA*

## Abstract

**Background:**

Food spoilage caused by molds is a severe problem. In food and feed, e.g. dairy products, sourdough bread and silage, lactic acid bacteria are used as starter cultures. Besides lactic and acetic acid, some strains produce other low molecular weight compounds with antifungal activities. One of these metabolites is phenyllactic acid (PLA), well known for its antifungal effect. The inhibitory effect of PLA has only partially been investigated, and the objective of this study was to elucidate in detail the antifungal properties of PLA.

**Results:**

We investigated the outgrowth of individual conidia from *Aspergillus niger*, *Cladosporium cladosporioides* and *Penicillium roqueforti*, and observed the morphologies of resulting colonies on solid media using different acid concentrations. We found that PLA inhibits molds similar to weak acid preservatives. Furthermore, it has an additional activity: at sub-inhibitory concentrations, fungal colonies displayed slower radial growth and inhibited sporulation. The L isoform of PLA is a more potent inhibitor than the D form. Increased expression of *phiA* was observed during PLA treatment. This gene was initially identified as being induced by *Streptomyces*-produced macrolide antibiotics, and is shown to be a structural protein in developed cells. This suggests that PhiA may act as a general stress protectant in fungi.

**Conclusion:**

From a food protection perspective, the results of this study support the usage of lactic acid bacteria strains synthesizing PLA as starter cultures in food and feed. Such starter cultures could inhibit spore synthesis, which would be beneficial as many food borne fungi are spread by airborne spores.

## Background

Spoilage of food and feed due to mold growth and synthesis of mycotoxins is a frequent problem which impacts health, economy and food security [1]. In some products, lactic acid bacteria (LAB) are used as biopreservatives and this may, to varying extents, prevent mold growth. For example, the bacterium *Lactobacillus plantarum* can be used as starter culture in dairy food products [2] and sourdough bread [3]. Moreover, LAB can be used as silage additives, based on the same principals applied when preserving food [4]. LAB can synthesize, in addition to lactic acid (LA), several low molecular weight molecules with inhibitory properties, e.g. organic acids, cyclic dipeptides, short peptides, hydroxylated fatty acids, and phenyllactic acid (PLA, both the D and L isomers) [5-7].

Many studies have investigated the antifungal activity of LAB strains producing PLA, in culture and model systems [8-10], and at the proteomic level [11]. However, apart from its pH-dependency, little is known about the actual antifungal mechanisms. However, D-PLA is reported to display antibacterial activity [12], and L-PLA is a competitive inhibitor of phenylalanine dehydrogenase in the bacterium *Rhodococcus* sp*.*[13]. To optimize the biopreservative effect of LAB, it has been suggested that bacterial strains with the greatest potential antifungal activity could be identified by screening for those producing high levels of PLA [14]. Moreover, PLA synthesis can be induced by adding phenylalanine [15]. In humans, PLA has been reported as an accumulated toxic product in patients suffering from the genetic disease phenylketonuria, but it has been shown to be non-toxic in cell lines [16].

One consequence of adding LAB strains as starter cultures is a reduction in pH. It is known that carboxylic acids, e.g. weak acid preservatives, LA, and some other LAB-produced metabolites, including PLA, have antimicrobial activity. According to the weak acid theory, at low pH the acid is uncharged and can thus cross the hydrophobic plasma membrane. The pH is higher inside the cell and the acid therefore dissociates, after which the charged anion cannot escape through the plasma membrane, causing stress by the increased activity required to maintain a correct intracellular pH. The trapped anion may additionally damage the cell by disrupting membranes, etc. There are several reports that weak acid preservatives, such as sorbic and benzoic acids, inhibit uptake of nutrients, e.g. amino acids [17-19] and nucleotides [20].

The aim of the present study was: to investigate the inhibitory properties of PLA; to reveal how it acts as a fungal inhibitor; and, to establish the concentrations required to display antifungal activity. We decided to primarily use the common food spoilage fungus *Aspergillus niger*, because it produces large numbers of asexual spores that are easy to extract, has a sequenced genome, and has been a target mold in other similar studies [9,20-22].

## Methods

### Strains and maintenance of fungal cultures

The fungal species and strains used in this study are listed in Table 1. Cultures were grown on malt extract agar (MEA; Oxoid) at 25°C. For the uridine auxotrophic *A. niger* strain AB4.1, all plates were supplemented with 10 mM uridine (Sigma-Aldrich). In all experiments, spores were harvested from 7-day old cultures.

**Table 1:** Fungal strains used in this study

**Species and strain**	**Origin/reference**
*Aspergillus niger*, N402	[23]
wildtype	
*Aspergillus niger*, AB4.1	[24]
Uridine auxotroph	
*Pencillium roqueforti*, J5	Dept of Microbiology, Swedish University of Agricultural Sciences
*Cladosporium cladosporioides* J308	Dept of Microbiology, Swedish University of Agricultural Sciences

### Statistical analysis

One- and two- way ANOVA with Bonferroni post test (Prism 5, GraphPad Software) were used to analyze statistical differences of the derived results.

### Preparation of agar plates supplemented with weak acids

The weak acids (all purchased from Sigma-Aldrich), DL-PLA (1:1 ratio), L-PLA acid, D-PLA, and benzoic acid were prepared by mixing each compound with lactic acid (VWR International). The acid mix was subsequently dissolved and sterilized in 1 ml ethanol (99%) before being added to autoclaved Aspergillus Complete Medium (ACM; per liter: NaNO_3_, 6 g; KCl, 0.52 g; MgSO_4_ · 7H_2_O, 0.52 g; KH_2_PO_4_, 1.52 g; FeSO_4_ · 7H_2_O, 0.5 mg; ZnSO_4_ · 7H_2_O, 0.5 mg; glucose, 2.0 g; casamino acids, 1.5 g; bactopeptone, 2.0 g; and yeast extract, 1.5 g). Immediately before pouring, the nutrient solution was mixed with autoclaved agar solution kept at 50°C, to give a final agar concentration of 1.5% w/v. For each acid blend, a total nutrient-agar solution of 100 ml was prepared and 80 ml were evenly distributed among four 90 mm petri dishes, with the remaining 20 ml used for pH measurement. For most assays, the total acid concentration was 150 mM (pH = 2.9). *A. niger* N402 DL-PLA was also tested on plates with 150 mM acid at pH 4.4 (adjusted with KOH prior to adding the agar). The acid blends comprised mixtures of LA and PLA, the latter typically added in increasing increments of 15 mM, i.e. starting with 15 mM PLA and 135 mM LA, up to 90 mM PLA and 60 mM LA. However, for *Penicillium roqueforti*, PLA was mixed in increments of 10 mM, up to 40 mM PLA. For benzoic acid, the first blend comprised 0.75 mM/149.25 mM LA, with increasing increments of 0.25 mM up to 3.0 mM. For *A. niger* AB4.1 and *Cladosporium cladosporioides* J308, the total acid concentration of LA and PLA was set at 100 mM (pH = 3.5), with increments of 10 mM PLA up to 40 mM.

### Spore preparation, colony morphologies and estimations of minimal inhibitory concentrations

Spores were harvested by adding 0.15 M NaCl containing 0.2% of Tween 80 to fungal colonies on agar plates. Next, the conidial suspensions were diluted in 0.15 M NaCl, counted in a Bürker chamber, and finally adjusted to 250 spores in 1 ml 0.15 M NaCl. 100 μl of spore suspension, i.e. 25 spores was evenly spread on ACM agar plates (in four replicates) containing different acid blends. Spores were also spread on ACM control plates containing either 100 mM or 150 mM LA, and on MEA plates.

The inoculated agar plates were incubated at 25°C for 14 days. The colonies were examined and counted after 3, 7 and 14 days to estimate the minimal inhibitory concentrations (MIC), here defined as when no colonies were noted during a thorough macroscopic observation. Any differences in colony growth and morphologies were also visually observed and recorded.

### Radial growth and spore count

The radial growth of single *A. niger* N402 colonies was measured on ACM plates containing 15 mM DL-PLA/135 mM LA and on ACM control plates with 150 mM LA, centrally inoculated with approximately 10 spores and incubated at 25°C for 3 and 7 days. The sporulation densities on the same plates were estimated by removing a circular area of 95 mm^2^ (11 mm diameter) from the center of the colonies. Samples were vortexed with 2 ml of 0.01% Tween80 and 10 glass beads (2 mm in diameter) for 10 min before counting the spore concentration in a Bürker chamber. Measurements of radial growth and sporulation were conducted on three biological replicates with three technical replicates each.

### RNA extraction, cDNA synthesis and real-time PCR

To extract total RNA, fungal spores were inoculated on plates covered with membrane filters (mixed cellulose esters, Metricel®, Pall Corporation, Michigan). After incubation for 3 and 7 days, the filters with growing colonies were transferred to mortars containing liquid nitrogen and ground to a fine powder with the pestle. Further extraction, cDNA synthesis, quantitative real-time PCR and calculations were performed essentially as described previously [25]. The real-time PCR protocol comprised the following steps: initial denaturation and enzyme activation 95°C 30 s; 40 cycles of 95°C 2 s and 56-60°C for 8 s; plate read, and finally, melt curve analysis starting at 65°C and ending at 95°C. The primers used are listed in Table 2. All results were calculated from three biological replicates with three technical replicates.

**Table 2:** Primers used in this study

**Primer name**	**Sequence 5′– 3′**	**Purpose/accession number**
T18r	TTTTTTTTTTTTTTTTTTVN	cDNA synthesis
actF	TCGTGACCTGACGGATTACCTC	qPCR actin
actR	TGGAAGAAGGAGCAAGAGCAGTG	ANI_1_106134
treBF	TGGACACTTACCTCTGGGATGAAG	qPCR *treB*
treBR	GCTGATGGGCATTGAGTATTTCC	ANI_1_1258014
tppAF	TTGAAGACACCGTTGGGAAGAG	qPCR *tppA*
tppAR	GGAGCAAAAGATGAACTCAGGAGC	ANI_1_1432094
phiAF	AGTTCGGTGGAAAGGACCTG	qPCR *phiA*
phiAR	ATACCCTCACAGCCCTCGTTGTAG	ANI_1_280124
brlAF	TGGGCAATGTGATACCTTG	qPCR *brlA*
brlAR	AATGAGCAGGAACGCACTGGAG	ANI_1_2984014
vosAF	TACGGAATCCCATCCTCCAC	qPCR *vosA*
vosAR	GTCTGGTATTGCGAACGAG	ANI_1_952184

## Results

### Assay setup and growth inhibiting properties of phenyllactic acid

In preliminary experiments examining the inhibitory effect of PLA at sublethal concentrations on wildtype *A. niger* spores, we found that fungal colonies grew more slowly and sporulation was inhibited (data not shown). However, increased concentrations of PLA in the culture medium resulted in a decreased pH. Accurately adjusting the pH with HCl to be equivalent to the highest PLA concentration tested was not possible since agar is rapidly destroyed if kept at acidic pH over a period of time and adding HCl just before pouring the plates resulted in too large variation in the final pH-value. Therefore, to obtain a constant pH in the following experiments, we used a mix of PLA with the potentially less inhibitory LA. Total PLA/LA concentrations of 100 mM and 150 mM resulted in pH values of 3.5 (+/− 0.05) and 2.9 (+/− 0.05), respectively, and there were no measurable differences when changing the relative concentrations of the two acids, despite the slight difference in their p*K*_a_ values (3.86 for LA and 3.72 for L-PLA).

**Figure 1:**
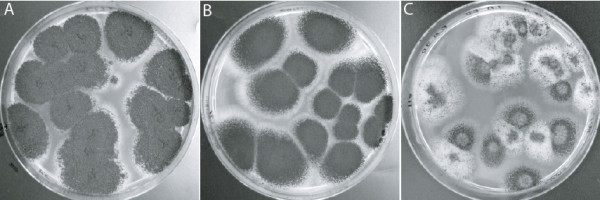
**Morphology of *A. niger *on control plates and with PLA.** Colonies grown 7 days on 90 mm petri dishes. Each plate was spread with approximately 25 conidia. Control on MEA **(A)**. Control on 150 mM LA, pH 2.9 **(B)**. Spores assayed with 90 mM PLA and 60 mM LA at pH 4.4 **(C)**.

**Figure 2:**
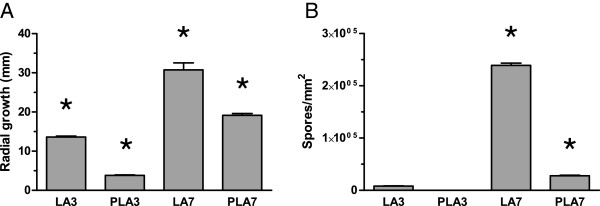
**Outgrowth and sporulation of single *A. niger *colonies assayed with PLA.** Colony diameter in mm with 150 mM LA and 15/135 mM PLA/LA after 3 and 7 days **(A)** and spore densities of the same colonies expressed as spores per square mm **(B)**. Error bars show standard error of the mean. The asterisks indicate values that were significantly different to all other values (p < 0.05).

**Figure 3:**
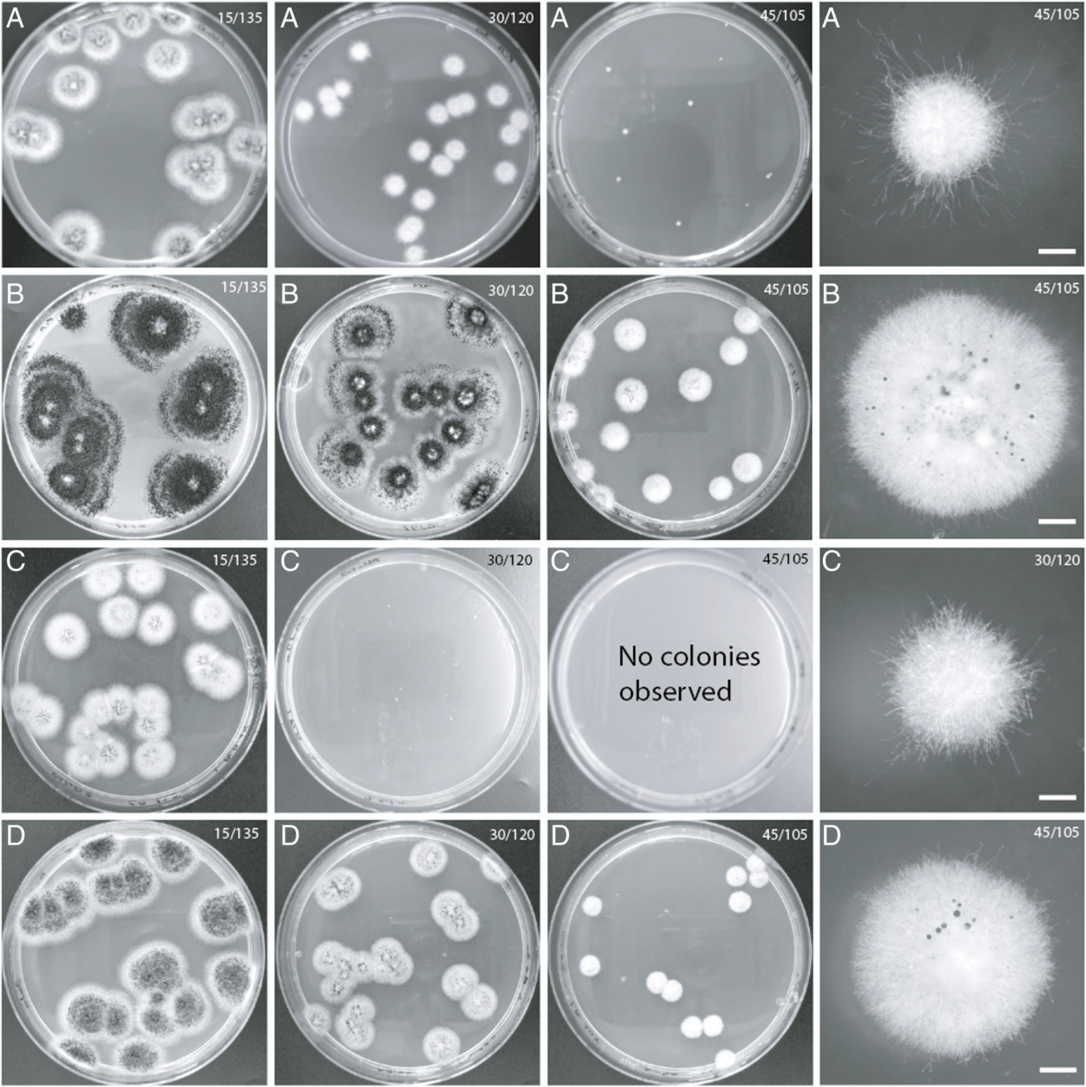
***A. niger *colonies grown at different concentrations of PLA and LA.** Pictures show 90 mm petri dishes. Concentrations of PLA and LA in mM are given at the top right corner of each subfigure. Scale bars where present = 1 mm. Photos taken after 7 days **(A)** and after 14 days **(B)** of spores assayed with a 1:1 DL-PLA mixture, L-PLA after 7 days where no growth was observed at 45 mM **(C)**, and D-PLA after 7 days **(D)**.

Using this experimental approach, we found that the growth and colony morphologies on control plates with 150 mM LA were generally identical to controls on MEA (Figure 1A-B). At pH 4.4 and a DL-PLA/LA concentration of 90/60 mM, the radial growth was similar to the two controls but sporulation was delayed in some colonies (Figure 1C, assayed visually). In contrast, the same acid blend at pH 2.9 (namely DL-PLA at 60 mM) was sufficient to totally repress growth of *A. niger*, i.e. the MIC was 60 mM. After 3 days incubation at 25°C with 15 mM of DL-PLA, radial growth was affected, and this was even more pronounced after 7 days when an apparent reduction in sporulation was also observed (Figure 2A and B, numerically assayed). Higher levels of sub-inhibitory concentrations, i.e. 30 and 45 mM of DL-PLA affected the radial growth and reduced the sporulation in a concentration dependent manner (Figure 3A, assayed visually). After 14 days, the MIC had not changed but sporulation had been initiated at lower acid concentrations. However, at 45 mM DL-PLA, sporulation was still limited and the colony sizes were smaller compared to the control (Figure 3B, assayed visually). Sub-inhibitory concentration of DL-PLA did not appear to decrease the number of colonies formed (Figure 3A). Note that morphologies of the controls were always observed after 3 days of incubation, as at later stages the plates were overgrown.

In order to check whether the two isoforms of PLA, i.e. the L and D forms, differed in activity, we repeated the test but using the two forms separately. The MIC values after both 7 and 14 days were as follows: 60 mM for D-PLA and 45 mM for L-PLA. When we examined the colonies under a stereomicroscope after 7 days of incubation on DL and L-PLA, we could only observe undifferentiated hyphae (Figure 3A,C). A few individual conidiophores could be observed on these colonies after 14 days, and on D-PLA plates after just 7 days (Figure 3B,D).

### Shared features between phenyllactic acid and weak acid preservatives

**Figure 4:**
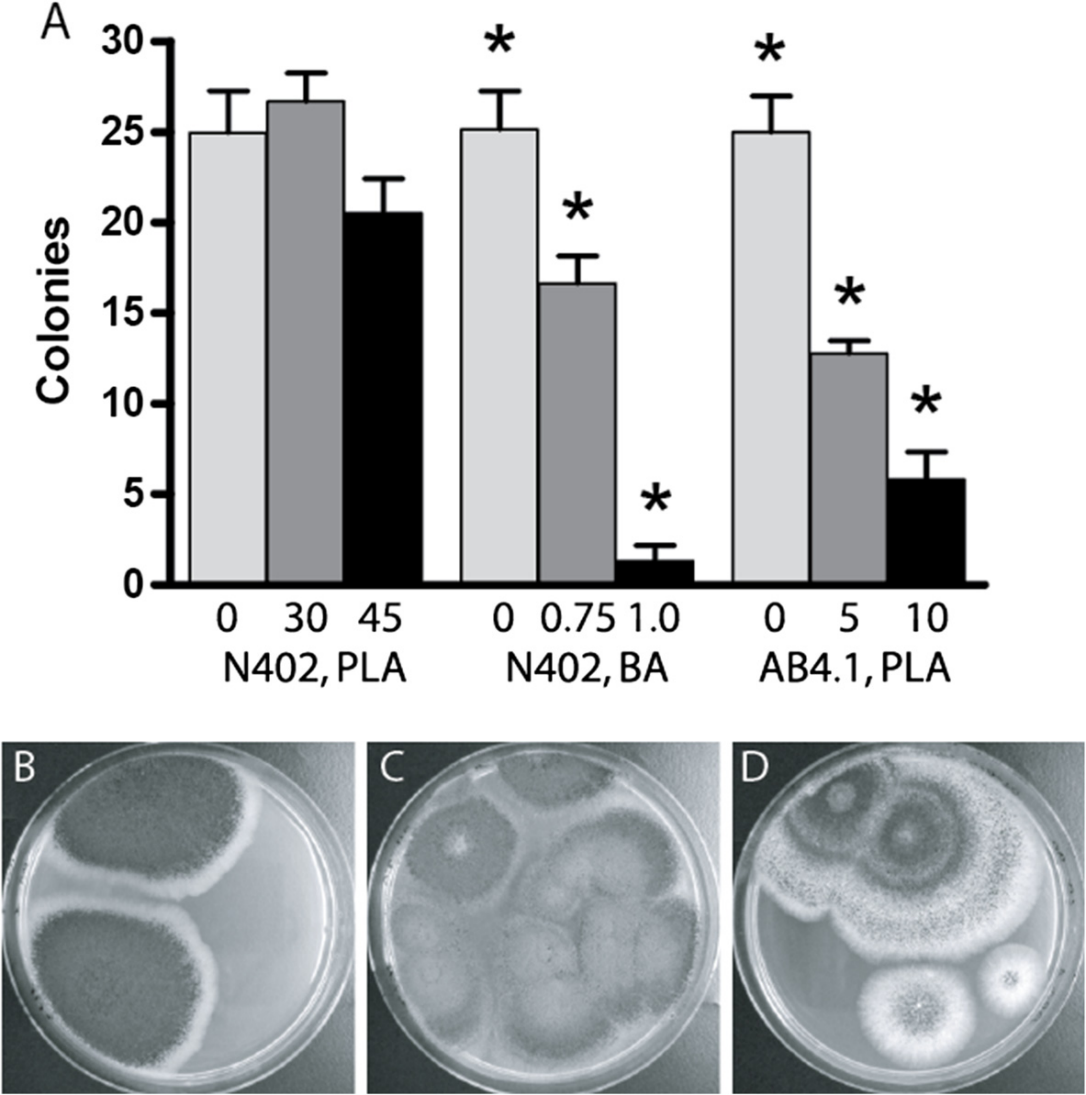
**Colony survival and morphology of *A. niger *wildtype and AB4.1 assayed with PLA and BA.** The graph **(A)** shows colony survival under acid stress, the concentration (mM) of DL-PLA or BA is given on the *x*-axis. For wildtype strain N402, LA was added to a total acid concentration of 150 mM, and for AB4.1, LA was added to 100 mM. The *y*-axis represents the average number of observed colonies (from 4 replicates, normalized to an average of 25 colonies in the controls) after 3 days (controls) and 14 days (assayed samples). Error bar show standard error of the mean. The asterisks indicate colony numbers that were significantly different to all others in the same group (p < 0.01). Morphologies of N402 colonies incubated for 14 days on 90 mm petri dishes assayed with 1 mM benzoic acid and 149 mM LA **(B)**, the uridine auxotroph strain AB4.1 on control plate with 100 mM LA **(C)**, and AB4.1 assayed with 10 mM DL-PLA and 90 mM LA **(D)**.

It could be argued that traditional weak acid preservatives in combination with LA affect colony growth and sporulation in a similar manner to PLA. To test this, we subjected *A. niger* wildtype N402 spores to benzoic acid (BA) that similar to PLA also contains a phenyl group. After 7 days of incubation, the MIC value of BA was 1.0 mM and after 14 days it was 1.5 mM. In contrast to PLA, we did not observe any changes in growth rate or sporulation of the growing colonies. Moreover, besides being effective at much lower concentrations compared to PLA, BA reduced the total number of colonies even at sub-inhibitory concentrations (i.e. below the MIC; Figure 4A-B), indicating a fungicidal activity.

It has previously been reported that uridine and adenine auxotrophs of *Aspergillus niger* were oversensitive to all tested weak acids, and that inhibited nutrient uptake was the likely mechanism [20]. To determine if PLA has this activity, we repeated our assay with PLA, but replaced the wildtype with a uridine auxotroph *A. niger* strain (AB4.1). The mutant was very sensitive and did not grow on the 150 mM LA control plates; therefore, we instead used a total acid concentration of 100 mM. In the control, the mutant strain grew similar to the wildtype controls, but addition of DL-PLA (10 mM PLA and 90 mM LA) resulted in fewer spores germinating compared to the control (Figure 4A,C-D), although the colony morphologies mostly resembled the control (Figure 4C-D). The MIC was estimated to be 15 mM in presence of 85 mM LA. Under these conditions, the wildtype strain N402 was unaffected both in number of viable colonies and colony morphology (data not shown).

### Gene expressions of *Aspergillus niger* assayed with phenyllactic acid

**Figure 5:**
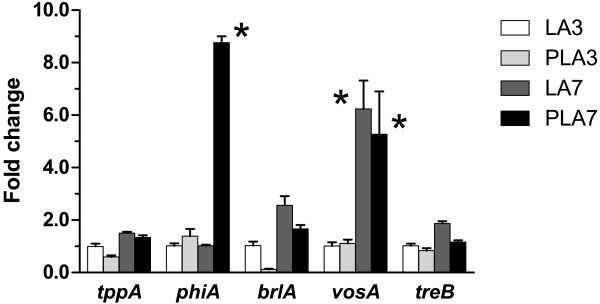
**Relative expression of *tppA*, *phiA*, *brlA*, *vosA *and *treB *in *A. niger *assayed with LA and PLA.** The concentrations were 150 mM LA and 15/135 mM PLA/LA. Gene expressions were analyzed after 3 and 7 days using actin as an internal standard. For all genes, the average expressions observed for LA3 were normalized to 1. Error bars show standard error of the mean. The asterisk indicates expressions that were significantly up-regulated (p < 0.0001) compared with the treatments in the same group.

To elucidate the effects of PLA compared to LA on sporulation, the expression of five genes known to be important for asexual reproduction in Aspergilli were examined. These genes were: *phiA*, induced by V-ATPase inhibitors [26-28] and essential for phialide development [29]; *tppA*, the *A. niger* tps2 ortholog encoding trehalose phosphate phosphatase [30], also essential for normal sporulation in *Aspergullus fumigatus*[31]; *vosA*, which is important for conidial maturation and also functions as a negative feed-back regulator of sporulation [32]; *treB*, the gene encoding for intracellular trehalase, which has been shown to be involved in spore production and viability [25]; and *brlA*, which regulates conidiophore stipe development [33]. All tested genes were expressed under all conditions, except for *brlA* in 3-day PLA cultures (Figure 5). The expression of *phiA* was up-regulated 9 fold in PLA 7-day cultures compared with the LA control after 7 days, and compared with both conditions after 3 days. The expression of *vosA* was up-regulated 6-fold in the 7-day cultures compared with the 3-day cultures for both LA and PLA. There were no significant differences in gene expression among the other genes under any condition.

### Inhibitory effects of phenyllactic acid tested on two other filamentous fungi

**Figure 6:**
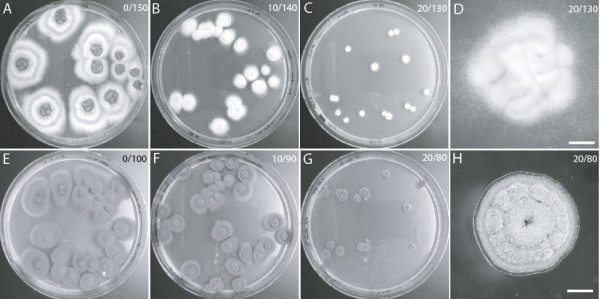
**Two additional fungi assayed with DL-PLA.** Concentrations of PLA and LA in mM are given at the top right corner of each subfigure. Scale bars when present = 1 mm. *P. roqueforti***(A-D)**, 90 mm petri dishes **(A-C)** and a single colony **(D)**. Sporulation was only observed in the LA control **(A)**. *C. cladosporioides***(E-H)**, 90 mm petri dishes **(E-G)** and a single colony **(H)**.

To widen the study and to determine if the observed effect in *A. niger* is common to other fungi, we repeated the test with DL-PLA on two other spoilage molds: *Penicillium roqueforti,* which is a frequent spoilage organism on cheeses, bread, stored cereal grains, etc.; and *Cladosporium cladosporioides*, which often spoils fruits and wheat. Similar to *A. niger*, both these species produce large numbers of spores enabling the same experimental approach. The MIC for DL-PLA on *P. roqueforti* was estimated to be 25 mM, and at 20 mM, a similar morphology to that of *A. niger* at sub-inhibitory concentrations of PLA was observed, namely restricted growth and poor conidiation (Figure 6). *C. cladosporioides* was not able to grow on the control plates with 150 mM LA at pH 2.9. Therefore, we modified the conditions for this species to those used for AB4.1, i.e. a total acid concentration of 100 mM and pH 3.5. Under these conditions, we calculated the MIC to be 30 mM, and at 20 mM, radial growth was reduced. However, in this fungus the sporulation was unaffected compared to the control (Figure 6).

## Discussion

In this study we have revealed previously unknown inhibitory modes of action of the *Lactobacillus*-produced metabolite PLA. We found that PLA at concentrations close to the MICs reduced the growth of all three tested molds and inhibited sporulation of *A. niger* and *P. roqueforti*. Notably, *Cladosporium* does not produce the same advanced conidia-producing structures, conidiophores, as *Aspergillus* and *Penicillium*[34]. Consequently, the results indicate that PLA inhibits hyphal differentiation rather than conidial syntheses *per se*; this was also supported by the gene expression results, discussed in detail below. This inhibitory mode was not observed for the structurally-similar weak acid preservative, benzoic acid. In contrast, benzoic acid was a stronger fungal inhibitor (with lower MIC) and displayed a higher level of fungicidal activity compared to PLA. However, the apparent lack of fungicidal activity may not be a problem, as in dairy products, and silage for animal feed, PLA-producing bacteria are viable and may be able to synthesize PLA constitutively, providing a continuous protection from spoilage fungi even if the compound is consumed or degraded by the spoilage mold.

Our results from the assay of the uridine auxotroph AB4.1 with PLA – the MIC value was lower and fewer spores germinated – indicate that at any concentration, PLA acts in a similar manner to other weak acids by inhibiting nutrient uptake [20]. Furthermore, as it is a rather large molecule, the inhibitory activity is greater compared to that of smaller molecules such as LA. At higher concentrations, PLA starts to influence the fungus by reducing growth and sporulation. In the oversensitive AB4.1 mutant, only the weak acid mode is active, as the spores were totally prevented from germination at a PLA concentration insufficient to cause the specific morphological changes. The hypothesis that the PLA main mode of function is the same as for other weak acids is further supported by the assay at high pH. When the external pH is low, weak acids exist in their undissociated form, and being lipophilic, they are able to cross the fungal plasma membrane [21]. At pH 4.4, colony sizes or number were not reduced in *A. niger* cultures at 90 mM PLA compared to the control, indicating that the function of PLA is pH-dependent in a weak acid manner.

Minimal inhibitory concentrations of PLA have been reported previously [9,10]. However, in this study we could also show that L-PLA is the most potent inhibitor, although D-PLA has a similar, but lower, activity. This observed difference between the isoforms contradicts previous findings [9]. However, besides estimating MIC-values with smaller increments in concentration, we monitored the outgrowth of individual conidia instead of observing growth in liquid media with high spore inoculums. Our approach has particular relevance in the food spoilage context, as mold spoilage often starts when one single airborne conidium lands on the surface and germinates. It has been reported that sub-inhibitory concentrations of preservatives can induce mycotoxin production [35], and this could be an argument against the use of PLA in food or feed. However, in that study, asexual development was not suppressed, and it has also been reported that there is a strong correlation between sporulation and toxin synthesis in molds [36]. The introduction of PLA-producing LAB strains to food and feed would hypothetically reduce sporulation, and, thus, may also suppress rather than stimulate mycotoxin synthesis. However, this hypothesis is likely dependent on mold species and possibly the target food, and requires further investigation. Compared to other preservatives, a relative high concentration of PLA is required to prevent growth. However, even at concentrations lower than the MIC, reductions in growth, and, for some molds, also the number of spores produced, would contribute to the overall antifungal activity, particularly in combination with other small molecules produced by LAB. This assumption is supported by a previous observation the in sourdough bread started with the PLA-producing *L. plantarum* strain 21B, growth of *A. niger* was delayed for 7 days compared to bread started with *L. brevis,* which do not produce PLA [37].

Expression analysis of a few selected genes involved in conidial development and, for some, possibly also stress protection, yielded some interesting trends. The increased expression of *vosA* in colonies grown for 7 days with both LA and PLA is in accordance with the findings of Ni and Yu for *A. nidulans*[32]: after induction of asexual development, the expression of *vosA* was elevated. This probably reflects the *vosA* negative feedback on sporulation, and is active to a similar extent in both LA and PLA cultures. However, the number of produced spores in PLA cultures after 7 days was significantly lower than in LA cultures (see Figure 2B), which might suggest that *vosA* is not activated by sporulation *per se* but rather the age of the colony or some other factor. The delayed sporulation in PLA cultures is in keeping with the fact that *brlA* expression (governing stipe development) was not observed in PLA 3-day cultures. There were no significant differences in the expression of *tppA* or *treB* between the LA and PLA assays, indicating that the sporulation-inhibiting properties of PLA are not coupled to trehalose accumulation or breakdown.

The expression of *phiA* after 7 days with PLA was strongly up-regulated compared to the expression after 3 days with PLA and in the LA controls (both 3 and 7 days). In *A. nidulans* the encoded protein, PhiA, is mostly present in phialides and in the conidia, and, based on sequence similarities, is proposed to be located in the cell wall. As a consequence, the *A. nidulans* deletion mutant failed to develop normal phialides and the number of conidia was reduced [29]. Moreover, *phiA* was initially identified as a gene induced by the *Streptomyces*-produced inhibitors of V-ATPases, bafilomycin and concanamycin, in *Aspergillus nidulans*[26,27] and later also shown in *A. niger*[28]. It is intriguing that the small LAB-produced molecule PLA induces the same gene as the large macrolide antibiotics produced by *Streptomyces* sp. This may indicate that PhiA, besides its role in asexual structures, is a general stress protectant produced by the fungus in competition with antagonistic bacteria.

## Conclusions

In addition to a broadened knowledge of fungal-bacterial interactions, the results derived from this study strongly support the usage of LAB strains synthesizing PLA as starter cultures in food and feed. Moreover, examining outgrowth of individual conidia on solid media has certain advantages over assays in liquid media with a large number of spores, namely, changes in radial growth rate and colony morphology can be observed. The dispersal of many common spoilage molds is dependent on airborne spores; therefore, inhibited sporulation induced by PLA potentially reduces fungal contamination, in addition to inhibiting growth.

## Abbreviations

PLA: Phenyllactic acid; LAB: Lactic acid bacteria; LA: Lactic acid; MEA: Malt extract agar; ACM: Aspergillus complete medium; MIC: Minimal inhibitory concentration; BA: Benzoic acid.

## Competing interests

The authors declare the absence of competing interests.

## Authors’ contributions

ÅS and SB contributed equally to the laboratorial work. EB also contributed to the laboratorial work. ÅS and PM conceived and designed the study and wrote the manuscript. All authors read and approved the final manuscript.
